# AHR Agonist ITE Boosted PD1 Antibody’s Effects by Inhibiting Myeloid-Derived Cells Suppressive Cells in an Orthotopic Mouse Glioma Model

**DOI:** 10.3390/ph18040471

**Published:** 2025-03-27

**Authors:** Pei Gong, Lijiao Zhao, Yunlong Ma, Qiuting Shu, Hui Sun, Jing Lu, Fanhua Meng, Fang Wan

**Affiliations:** Tumor Molecular Pharmacology Laboratory, College of Life Sciences, Inner Mongolia Agricultural University, Hohhot 010011, China; gongpei@imau.edu.cn (P.G.); 18248114094@163.com (L.Z.); 13404808590@163.com (Y.M.); shuqiuting@outlook.com (Q.S.); sh15248190086@126.com (H.S.); lujing185040@126.com (J.L.)

**Keywords:** glioblastoma, ITE, AHR, PD1, IL11

## Abstract

**Background:** Glioblastoma is “cold”. Consequently, immune checkpoint blockade therapy has failed to improve patients‘ survival, which is negatively correlated with patients’ peripheral MDSC counts. AHR is known to mediate immune-suppressive functions of certain tryptophan metabolites such as kynurenine; yet, there lack of reports on how AHR agonists affect glioma immunity. **Methods/Objectives**: We hypothesized that ITE could synergize with PD1 antibody as AHR is one major node of immune-suppressive pathways, and tested it using an immune-competent mouse glioma model. **Results**: The combination of ITE+PD1 antibody glioma MDSC was significantly reduced, along with increased infiltration of the CD4−CD8+ and CD4+CD8+ T cells, leading to extended mouse survival. ITE treatment alone significantly reduces the infiltration of CD11b+Ly6G+Ly6Clo cells, namely PMN-MDSCs, and neutrophils, while the combination with PD1 antibody significantly reduces all MDSCs plus neutrophils. The presence of ITE inhibits the expression of IL11 and the in vitro induction of MDSCs from mouse PBMCs by IL11. The identification of the ITE-AHR-IL11-MDSC pathway provides more mechanistic insights into AHR’s effects. The fact that ITE, which is otherwise immune-suppressive, can activate immunity in glioma indicates that searching for drugs targeting AHR should go beyond antagonists.

## 1. Introduction

Immunotherapy for glioblastoma (GBM) has been challenging due to the predominant “cold” tumor phenotype with reduced immune cell infiltration, despite increased immune cell infiltration overall compared to normal brain tissue [1]. Systematic immune function is inhibited in both GBM patients and mouse orthotopic models, leading to impaired T cell function and increased myeloid-derived suppressor cells (MDSCs) [2,3,4]. In GBM patients, T cells are also sequestered in the bone marrow from peripheral blood [5].

GBM cells express proteins on their surface that directly inhibit cytotoxic immune cells, such as HLA-G and HLA-E for natural killer (NK) cell inhibition, and PD-L1 for inhibition of CD8+T cells [6]. Additionally, GBM cells release immunosuppressive proteins and molecules into the tumor microenvironment, such as kynurenine and TGF-β [7,8], as well as exosomes containing PD1 protein. Kynurenine, a tryptophan metabolite generated by IDO, has been detected in glioblastoma specimens and is significantly associated with poor prognosis [9]. Moreover, IFN-γ can induce IDO expression [10], which mediates adaptive resistance of tumors to PD-1/PD-L1 or CTLA-4 checkpoint blockades [11].

Overcoming the highly suppressive GBM microenvironment requires activating both innate and adaptive immunity, and mere checkpoint blockade is insufficient. Although anti-PD1 antibody nivolumab was in a phase III clinical trial in GBM patients, preliminary results did not meet the endpoint, with median overall survival in the nivolumab treatment group similar to the bevacizumab-treated control group [12].

One major pathway leading to resistance to PD1 blockade is the IDO/TDO-kynurenine-AHR pathway. In patients with glioblastoma, the kynurenine/Trp ratio is a prognostic factor for immunotherapy outcome [13]. However, clinical trials combining PD1 blockade with IDO/TDO inhibitors have not met expectations in other cancer types. It has been shown that IDO inhibition alone is not sufficient to overcome PD1 resistance in glioma, as adding radiation to an IDO1 inhibitor and PD1 blockade increases survival in an orthotopic murine glioma model [14]. Recently, it has been found that IDO/TDO is not the only immune-suppressive pathway that uses AHR. Another IDO/TDO-independent tryptophan metabolite, indole-3-aldehyde, binds to AHR and suppresses immunity [15].

Furthermore, ITE bound to AHR and inhibited the transcription of POU5F1 in glioma stem cells, which led to the inhibition of tumor growth in the U87MG xenograft glioma model. AHR has also recently been identified as a suppressor of lung metastasis [16]. We hypothesized that ITE could compete with immune-suppressive AHR ligands such as kynurenine and indole-3-aldehyde, and overcome PD1 antibody resistance.

In this study, we report that the combination of ITE and PD1 antibody treatment increased CD8+T cell infiltration in an orthotopic glioma model, likely through the inhibition of MDSCs, and extended mouse survival.

## 2. Results

### 2.1. ITE Synergizes with PD1 Antibody to Suppress Tumor Growth and Extend Animal Survival

**Figure 1:**
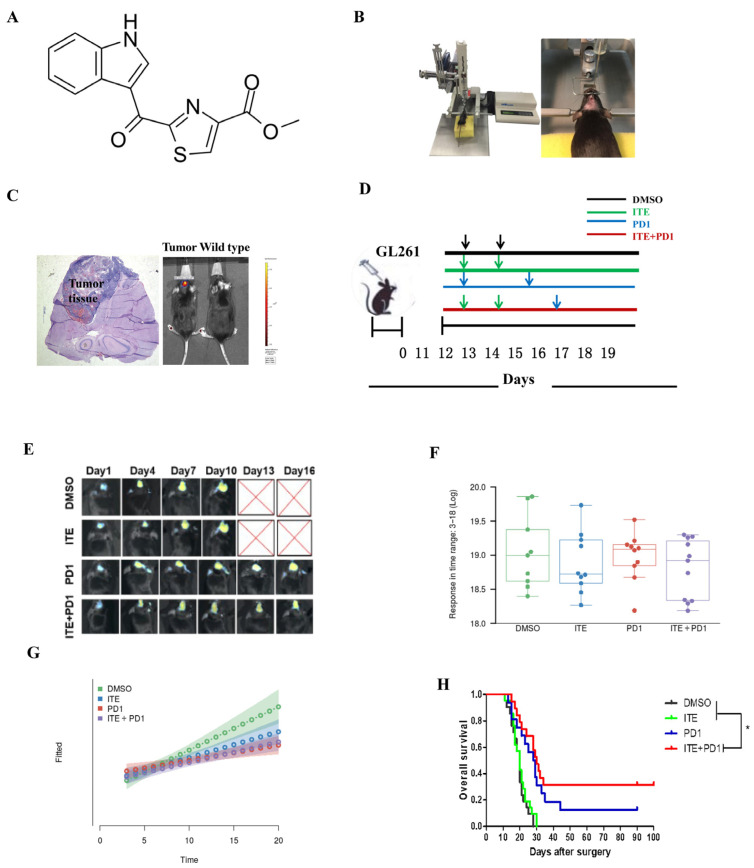
Synergistic effect of ITE and PD1 antibody on tumor growth suppression and animal survival: (**A**) ITE structure. (**B**) Tumor model was established by injecting 1 × 10^5^ mouse glioma cells GL261 into the brain of mice using a stereotaxic device. (**C**) Tumor growth in the model mice was confirmed by H&E staining of whole brain sections and by injecting the fluorescent dye IR783 (100 nmol/mouse) into the intraperitoneal cavity of mice. Fluorescent area indicated tumor enrichment. (**D**) Four treatment groups were given to the model mice: 10% DMSO/mouse, 100 mg/kg ITE/mouse, 80 μg PD1 antibody/mouse, and a combination of 100 mg/kg ITE and 80 μg PD1 antibody/mouse. (**E**) Post-treatment mice were intracranially injected with 100 µmol IR-783, and average tumor luminescence was determined using an IVIS spectrum imaging station. Representative IVIS spectrum images of treated mice with intracranial GL261 at days 0, 3, 6, and 9 post-intracranial injection treatment were obtained. (**F**) Tumor volumes were measured. (**G**) Tumor growth trends were analyzed using a linear mixed model. (**H**) Survival analysis was conducted on wild-type C57BL/6 mice that were intracranially injected with 1 × 10^5^ GL261 cells and received treatment with DMSO, ITE, PD1, or combination therapy of ITE with PD1, all starting 14 days post-intracranial injection (post-ic.) (*, *p* < 0.05).

To evaluate the anti-glioma effect of ITE (structure in Figure 1A), we established an orthotopic mouse glioma model by injecting GL261 cells intracranially and monitored tumor growth using in vivo imaging with a near-infrared fluorescent dye, IR783, which targets tumor cells specifically. Tumors became detectable on day 11 after implantation, which was designated as day 1 (Figure 1B,C). Animal body weights were monitored daily. Treatment with ITE, PD1 antibody, or a combination of ITE and PD1 antibody (designated as ITE+PD1) started on the first day of body weight drop (Figure 1D). Animals were observed daily and imaged every three days until they were euthanized when the posterior tumor edge approached the tragus of the ears for animal welfare.

We analyzed the imaging data using a linear mixed model fitted with the online tool TumorGrowth. The results showed that although the ITE+PD1 group had a trend of slower tumor growth compared to the DMSO control group (Figure 1F,G), the growth curve difference did not reach statistical significance (*p* > 0.05). However, the survival of mice in the ITE+PD1 group was significantly extended. Some survivors in the PD1 and ITE+PD1 groups were observed for 100–180 days after the euthanasia of all other animals and were found to be cured upon autopsy.

### 2.2. ITE Synergized with PD1 Antibody to Increase Cytotoxic Immune Cells Infiltration in Glioma

**Figure 2:**
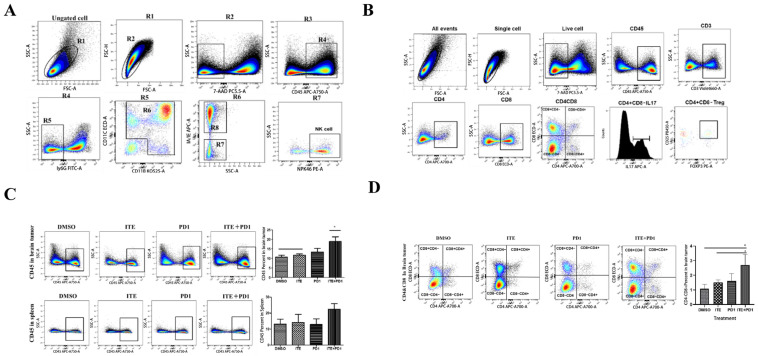

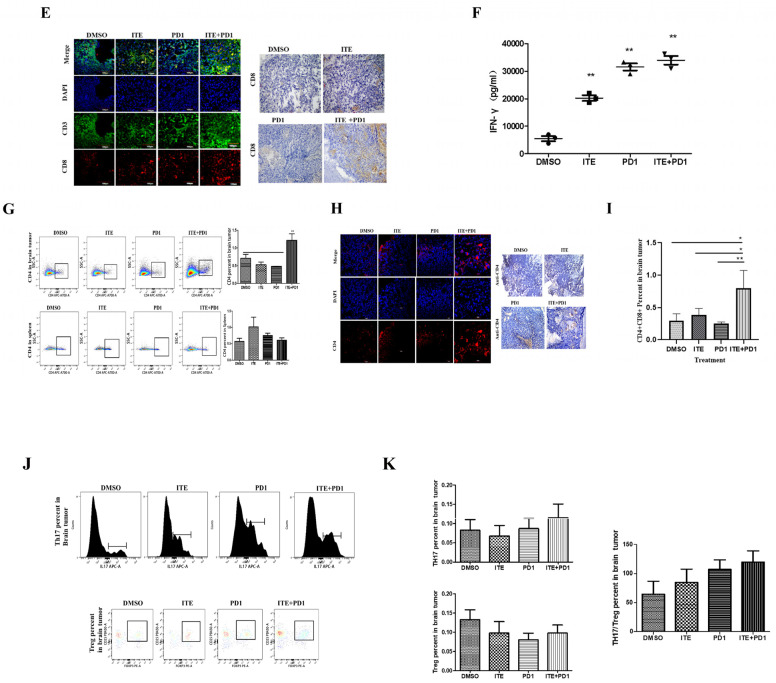
Synergistic effect of ITE and PD1 antibody on cytotoxic immune cell infiltration. (**A,B**) Gating Strategy for myeloid cells. R2 represents single cells, R3 represents live cells, R4 represents CD45+ immune cells, R5 represents non-neutrophils, and R6 represents myeloid cells. R7 was composed of CD11c+ lymphocytes, NK cells, and monocytes. (**C**–**G**) Effects of DMSO, ITE, PD1 antibody, and ITE+PD1 antibody treatments on immune cells in brain tumors and spleen. (**C**) Percentage of CD45+ cells in brain tumors and spleen (* *p* < 0.05). (**D**) Percentage of CD4-CD8+T cells in brain tumors and spleen (* *p* < 0.05). (**E**) Immunofluorescence and immunohistochemical staining of frozen brain tumor sections to detect CD8 T-cell antigens; blue: cell nucleus, red: CD8. (**F**) Detection of IFN-γ in brain by ELISA. (**G**) Percentage of CD4+T cells in brain tumors and spleen (** *p* < 0.05). (**H**) Immunofluorescence and immunohistochemical staining of frozen brain tumor sections to detect CD4 T-cell antigens; blue: cell nucleus, green: CD3, red: CD4. (**I**) Percentage of CD4+CD8+T cells in brain tumors (* *p* < 0.05, ** *p* < 0.01). (**J**,**K**) Th17/Treg cells in brain tumor.

Flow cytometry analysis was conducted on glioma and spleen tissues, with the gating and cell identification strategy shown in Figure 2A,B. The results revealed an increased percentage of CD45+ cells in the ITE+PD1 group, indicating a higher infiltration of immune cells in both glioma tissue and spleen (Figure 2C). Accordingly, the percentage of cytotoxic CD4-CD8+T cells was significantly higher in the ITE+PD1 group than in the DMSO and ITE groups (Figure 2D). Immunohistochemistry (IHC) and immunofluorescent (IHF) staining of glioma tissue confirmed this increase and further revealed that CD8+T cells infiltrated deeper into the glioma tissue upon ITE treatment, as shown in Figure 2E. To assess the function of the tumor-infiltrated CD8+T cells, the level of IFN-γ was determined and found to be significantly increased in the PD1 and ITE+PD1 groups (Figure 2F).

CD4+T cells were significantly increased (Figure 2G) in the glioma tissue of the ITE+PD1 group, which was further confirmed by immunostaining of the tissue (Figure 2H), indicating a local expansion rather than recruitment from the spleen. Further analysis of the Th17/Treg ratio revealed no significant changes across various groups. Interestingly, a CD4+CD8+T cell population was found to be significantly increased in the ITE+PD1 group, indicating that AHR also regulated this relatively unknown population (Figure 2D,I).

### 2.3. ITE Inhibited MDSC Infiltration in the Brain Tumor

**Figure 3:**
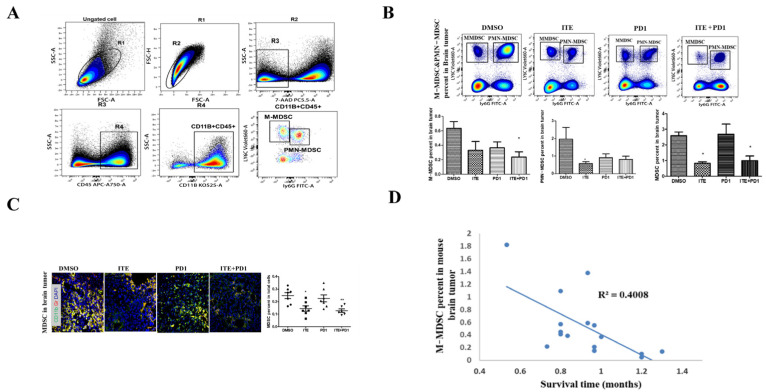
Effect of ITE on MDSC infiltration in brain tumor tissue. (**A**) Flow cytometry gating strategy for identifying PMN-MDSCs, M-MDSCs, and MDSCs. (**B**) Proportion of M-MDSCs, PMN-MDSCs, and total MDSCs in brain tumor tissue treated with DMSO, ITE, PD1 antibody, and ITE combined with PD1 antibody. (**C**) Immunofluorescence staining of brain tumor tissue for MDSC antigens, CD11B and Gr-1(binds to Ly6G and Ly6C), after treatment with DMSO, ITE, PD1 antibody, and ITE combined with PD1 antibody. Blue: cell nucleus; green: CD11B; red: Gr; merge: MDSCs. (* *p* < 0.05, ** *p* < 0.01). (**D**) The relationship between the ratio of MDSCs and survival of model mice.

MDSCs exhibit multiple immune-suppressive effects and are divided into CD11b + Ly6ChiLy6G- marked M-MDSCs and CD11b + Ly6CloLy6G+ marked PMN-MDSCs. As mouse neutrophils also exhibit a Ly6CloLy6G+ phenotype, we refer to these cells as PMN-MDSCs + neutrophils in this study. Flow cytometry analysis of the MDSCs was carried out, with a commonly used gating strategy (Figure 3A). The percentage of M-MDSCs was significantly reduced in the ITE+PD1 group (Figure 3B), while the percentage of PMN-MDSCs + neutrophils was significantly reduced in the ITE-only group (Figure 3B). As a result, the sum of M-MDSC and PMN-MDSC + neutrophil percentages was significantly reduced in both thr ITE and the ITE+PD1 group. Immunofluorescence analysis of glioma tissue using Gr-1 and CD11b antibodies confirmed this finding (Figure 3C).

Furthermore, the infiltration pattern of MDSCs was altered in the ITE+PD1 group, with MDSCs primarily located at the periphery of the glioma tissue compared to the DMSO or PD1 group. This change in the MDSC tissue infiltration pattern was in contrast to the CD8+T cell pattern. To provide a preliminary functional analysis of MDSC cells, we plotted MDSC percentage against mouse survival days and found a negative correlation between MDSC percentage and survival (r = 0.63) (Figure 3D), confirming the role of MDSCs in this model.

### 2.4. Kynurenine Induced STAT3, IL11 Expression Was Suppressed by ITE

**Figure 4:**
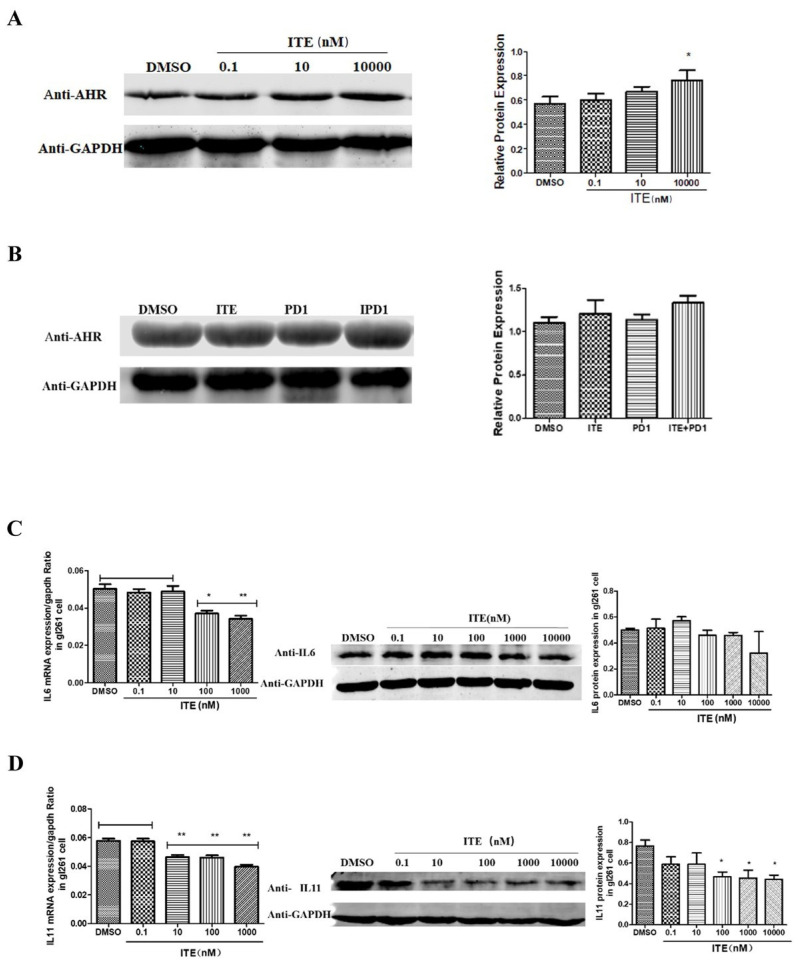

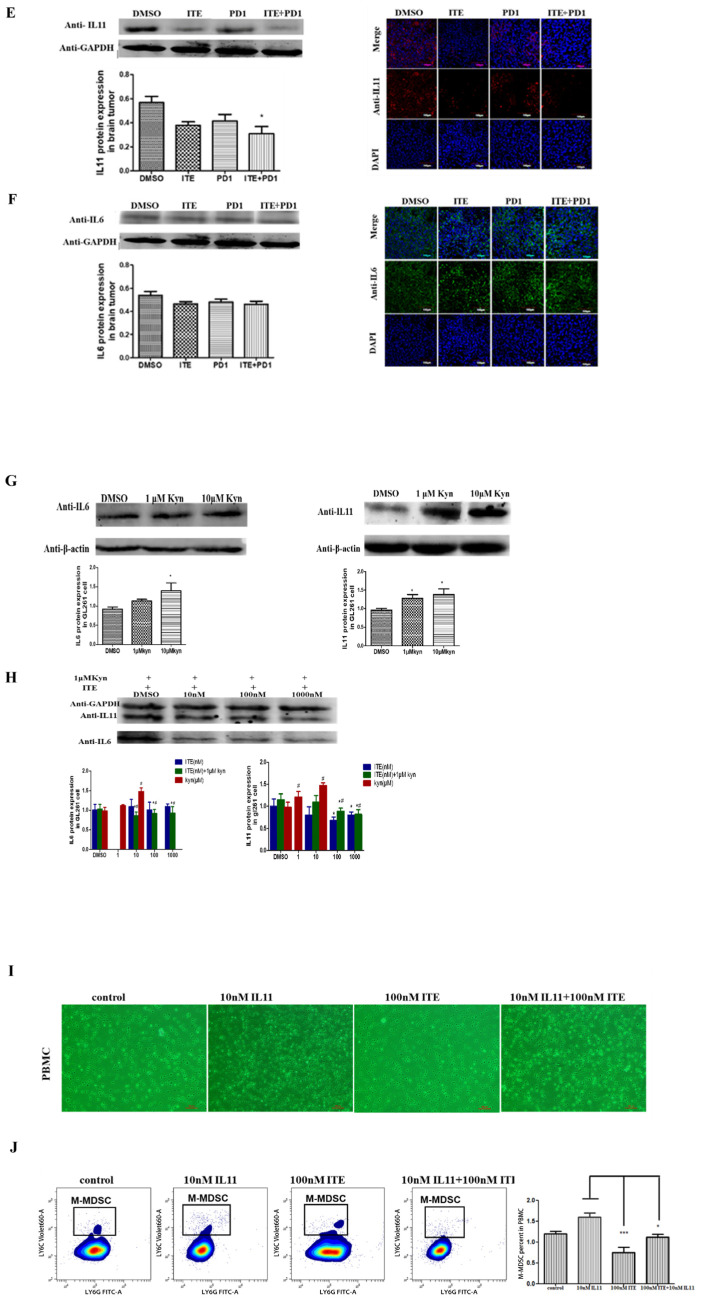
ITE treatment significantly down-regulated IL11 in GL261 cells. (**A**) Western blot showing the protein levels of AHR in GL261 cells treated with DMSO and ITE (0.1 nM, 10 nM, 10,000 nM) for 20 h (* *p* < 0.05). (**B**) Western blot showing the protein levels of AHR in brain tumors treated with DMSO, ITE, PD1 antibody, and the combination of ITE with PD1 antibody. (**C**,**D**) Q-PCR and Western blot showing the mRNA and protein levels of IL6 and IL11 in GL261 cells treated with DMSO or ITE (0.1 nM, 10 nM, 10,000 nM) for 20 h (* *p* < 0.05, ** *p* < 0.01). (**E**,**F**) Western Blot and immunofluorescent staining showing the protein level of IL11 and IL6 in brain tumors treated with DMSO, ITE, PD1 antibody, and the combination of ITE with PD1 antibody. DAPI stains the cell nucleus, green represents IL6, and red represents IL11 (* *p* < 0.05). (**G**) Western blot showing the protein levels of IL6 and IL11 in GL261 cells treated with kynurenine (1 μM, 10 μM) (**H**) Western blot showing the protein levels of IL6 and IL11 in GL261 cells upon treatment of 1 μM kynurenine combined with ITE (10 nM, 100 nM, 1000 nM) for 20 h (* denotes that the treatment group was significantly different from the DMSO group; # denotes that the treatment group was significantly different from the Kyn+DMSO group, */# *p* < 0.05. (**I**) PBMCs were collected from the blood of normal C57BL/6 mice and treated with IL-11 (10 nM) and ITE (100 nM), and the combination of 10 nM IL11 with 100 nM ITE for 5 days. The number and morphology of PBMCs were recorded under a microscope. (**J**) Flow cytometry was used to detect the proportion of M-MDSCs in PBMCs treated with DMSO, 10 nM IL-11, 100 nM ITE, and the combination of 10 nM IL11 with 100 nM ITE (* *p* < 0.05, *** *p* < 0.005).

Known to suppress immunity in experimental autoimmune encephalomyelitis (EAE), ITE’s immune-activating effects observed in our glioma model could be due to the blocking of other immune-suppressive agents such as kynurenine in the tumor microenvironment. To rule out the possibility of ITE’s effect depending on diminished AHR level, the AHR protein level in GL261 cells and glioma tissues was examined. The level was slightly increased upon ITE treatment of GL261 cells in vitro (Figure 4A) but remained unchanged in glioma tissues of all groups (Figure 4B), indicating that ITE exerts its effects via AHR.

We then tested whether ITE could block kynurenine’s induction of a known AHR target and MDSC regulator, IL6, and its family member IL11, as they were down-regulated by ITE according to our RNA-seq data (NCBI bioproject PRJNA788010 for U87MG, PRJNA789328 for GL261). ITE significantly down-regulated IL6 and IL11 mRNA levels in GL261 cells (Figure 4C,D). Moreover, IL11 protein levels were also reduced (Figure 4D). Unexpectedly, the IL6 protein level remained unchanged, indicating additional regulation in the post-transcription level. Accordingly, in mouse glioma tissue, IL11 level was inhibited in both the ITE and the ITE+PD1 group, with the ITE+PD1 group reaching statistical significance (Figure 4E). No significant changes were detected in the IL6 tissue (Figure 4F).

To test whether ITE can inhibit IL6 and IL11 expression in the presence of kynurenine, we first examined how kynurenine affected them. Kynurenine (1 μM, 10 μM) induced the expression of both IL6 and IL11 (Figure 4G), confirming that IL11 is also an AHR target. In the presence of 1 μM kynurenine, IL11 expression was inhibited by ITE (Figure 4H). Interestingly, while ITE alone did not significantly suppress IL6, kynurenine-induced IL6 expression could be suppressed by ITE, suggesting that ITE exerted significant blocking effects only in the presence of kynurenine, fitting a definition of antagonist.

IL11 was known to induce PBMC differentiation into MDSCs in vitro, and we tested whether ITE may reduce MDSCs in cultured PBMCs. ITE showed a trend of M-MDSC reduction, yet not reaching statistical significance. When IL11 was added to induce MDSCs, ITE significantly reduced the M-MDSC percentage. (Figure 4I,J) again shows a significant blocking effect only in the presence of a stimulant.

**Figure 5:**
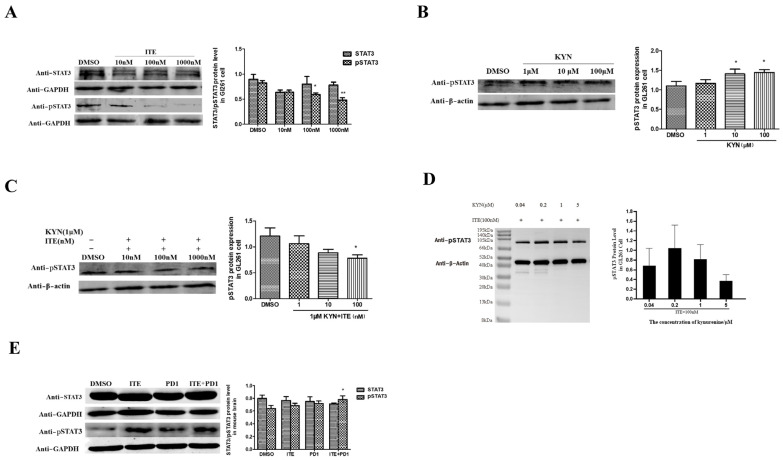
Effect of ITE treatment on pSTAT3 in GL261 cells and brain tumor tissue. (**A**) Western blot analysis of STAT3 and pSTAT3 protein levels in GL261 cells treated with DMSO and ITE (10 nM, 100 nM, 1000 nM) for 20 h (* *p* < 0.05, ** *p* < 0.01). (**B**,**C**) Western blot analysis of pSTAT3 protein levels in GL261 cells treated with kynurenine (1 μM, 10 μM, 100 μM) and a combination of 1 μM kynurenine with ITE (1 nM, 10 nM, 100 nM) for 20 h (* *p* < 0.05). (**D**) Western blot analysis of pSTAT3 in GL261 cells treated with 100nM ITE plus various kynurenine (0.04 μM, 0.2 μM, 1 μM, 5 μM). (**E**) Western blot analysis of STAT3 and pSTAT3 protein levels in brain tumor tissue treated with DMSO, ITE, PD1, and ITE+PD1 combination (* *p* < 0.05).

STAT3 is a key downstream effector of IL-6 and IL-11 that regulates MDSC function. Our findings demonstrated that ITE significantly down-regulated phosphorylated STAT3 (pSTAT3) levels in GL261 cells (Figure 5A), whereas kynurenine at concentrations of 10 μM and 100 μM increased pSTAT3 levels in a dose-dependent manner (Figure 5B). To investigate the interaction between ITE and kynurenine, we treated GL261 cells with ITE (1 nM, 10 nM, 100 nM) in the presence of either 1 μM or 10 μM kynurenine. With 10 μM kynurenine, extensive cell death occurred. However, with 1 μM kynurenine, pSTAT3 levels displayed a monotonic dose–response trend, with 100 nM ITE significantly inhibiting pSTAT3 levels (Figure 5C), suggesting that ITE acted as an antagonist. Conversely, we analyzed pSTAT3 levels following treatment with varying concentrations of kynurenine (0.04 μM, 0.2 μM, 1 μM, 5 μM) in the presence of 100 nM ITE (Figure 5D), which revealed that kynurenine elicited a biphasic dose–response trend, indicating a complex interaction between these two AHR ligands. Interestingly, glioma tissue displayed an increase in pSTAT3 levels upon ITE+PD1 treatment, suggesting that IL-11 and IL-6 are likely not the primary regulators of STAT3 in this context and highlighting STAT3 as a potential target for combination therapy (Figure 5E).

## 3. Discussion

AHR antagonists have been studied and developed extensively in recent years. For instance, CH223191 has been reported to restrict a Treg-macrophage suppressive axis and significantly suppress tumor growth in IDO over-expressing tumor models [17]. BAY 2416964 has also entered clinical trials after inhibiting tumor growth in mouse models [18]. Here, we found that ITE, an AHR agonist that suppressed immunity in EAE and experimental colitis [19,20], activated anti-tumor immunity when combined with PD1 antibodies, suggesting that the efforts for targeting AHR shall go beyond searching for antagonists.

In the orthotopic GL261 mouse model, immune profiling revealed that in the ITE+PD1 group, cytotoxic CD4-CD8+T cells infiltrated more and deeper into the glioma tissue [21,22]. CD8+T cell infiltration in GBM is positively correlated with the long-term survival of GBM patients [23]. Accordingly, more mice were cured by the combination therapy compared to the PD1 group. The increased IFN-γ also confirmed activated anti-tumor immunity. Additionally, we found a CD4+CD8+T population was induced by the ITE+PD1 treatment.

Little is known about the origin of this population, and the appearance of these cells in the ITE+PD1 group indicated that AHR could regulate their generation. The few functional studies of CD4+CD8+T cells reported either anti-tumor or antiviral activities: In the tumor model they were a heterogenous population with cytotoxicity and reactivity towards cancer cells [24]. DPT cells showed differentiated effector memory cells’ phenotypes with antiviral activities [25], or enhanced responses to IL-2, IL-7, and IL-15 [26]. Our finding that a large portion of CD8+T cells were actually DPT seems to also support an anti-tumor function of these cells.

For changes in the immune landscape, we monitored the CD4+T cell populations infiltrating the tumor, specifically the Th17 and Treg populations, as AHR is a master regulator of CD4+T cell development. TCDD directly modulates both CD4 + and CD8+T cells [27], while kynurenine suppresses the proliferation of T cells [28] and memory CD8+T cells [29]. In response to the PD1 antibody-induced IFN-γ increase, the IDO-kynurenine pathway inhibits CD8+T cells by controlling Treg differentiation to lower the Th17/Treg [30]. However, in our model, ITE+PD1 did not significantly change the Th17/Treg ratio, but activated immunity by down-regulating MDSCs.

The significant reduction in total MDSCs by ITE+PD1 could explain its ability to boost the effects of PD1 antibodies and extend animal survival. Peripheral blood of GBM patients showed higher levels of MDSCs compared to Tregs, and higher MDSC levels in recurrent tumors predicted poor prognosis in glioma [31]. MDSCs are reported to mediate PD1 resistance by inhibiting CD8+T and NK cells, so ITE’s MDSC inhibition effect might help overcome PD1 resistance.

ITE alone suppressed PMN-MDSCs + neutrophils, which could be a direct effect of ITE on AHR-expressing PMN-MDSCs. It is worth noting that for the glioma model, depletion of PMN-MDSCs extended survival only in female mice, while M-MDSCs were predominant in males [32]. As a common practice in glioma research, we used all-female mice in our study [33,34], and how ITE affected males needs to be examined in the future.

ITE could also indirectly regulate MDSCs’ mobilization and function. Another AHR ligand, TCDD leads to massive mobilization of MDSCs through the regulation of CXCR2, miR-150-5p, and miR-543-3p [35]. AHR-expressing oral squamous carcinoma cells induced the expression of checkpoint molecules PDL1 and CD39 in MDSC [36].

Although we monitored M-MDSCs and PMN-MDSCs in the spleen, our data are not sufficient to discern whether ITE affected the expansion, mobilization, or conversion/differentiation of MDSCs, as data on neutrophils and monocytes are lacking. Spleen Ly6C+ Ly6G− cells could be either M-MDSC or the newly identified PMN-MDSC progenitors, monocyte-like progenitors of granulocyte (MLPG) [37]. Further analysis with additional markers is required to clarify how ITE affected each MDSC species.

Given the opposing effects of ITE in autoimmune diseases and cancer models, we hypothesized that ITE might competitively block other ligands by binding to AHR with high affinity. Our findings indicate that ITE significantly inhibited kynurenine-induced expression of IL-6, IL-11, and pSTAT3 levels, providing support for this hypothesis. To further investigate the interaction between these two AHR ligands, cells were treated with varying concentrations of ITE in the presence of 10 µM kynurenine. However, these conditions resulted in extensive cell death. Interestingly, in the presence of 100 nM ITE, kynurenine-induced pSTAT3 exhibited a biphasic, hormetic-like dose–response curve. This behavior suggests a complex interplay between ITE and kynurenine, rather than a simple competitive inhibition, which would typically manifest as a monotonic dose–response curve that shifts upon the addition of an inhibitor.

Interestingly, a similar biphasic response has been observed with resveratrol, another AHR ligand [38], suggesting that this phenomenon may reflect an intrinsic property of AHR signaling or arise from the interaction of multiple ligands. Notably, AHR signaling is unique in that ligand binding induces CYP1A1 expression [39], which may degrade the ligand. It is plausible that certain AHR ligands illicit drug effects, such as pSTAT3 signaling, while simultaneously inducing CYP1A1 expression. At higher doses, increased CYP1A1 activity could degrade the ligand, thereby attenuating the drug’s effects at elevated concentrations. The mechanisms underlying these hormetic biphasic responses warrant further investigation.

IL11 has not been previously associated with AHR but is known to suppress CD4+T cells mediated anti-tumor immunity, and the IL11/STAT3 inhibition increased MHC-I expression and T cell infiltration in colon cancers [40,41]. Our discovery of the opposite regulation of IL11 by ITE and kynurenine suggests that it is an AHR target in glioma.

As expected, ITE treatment reduced IL6/IL11 in cultured glioma cells. Unexpectedly, overall STAT3 phosphorylation level was increased in the ITE+PD1 group of glioma tissue, indicating that glioma cells might respond to other upstream factors of STAT3 that exist in tissue but not in the cell culture medium or non-cancerous cells such as macrophages/microglia might respond to ITE by activating STAT3. The activated STAT3 in the ITE+PD1 group suggests that STAT3 inhibitors shall be explored as an addition to the current combination reported here, specifically in those gliomas with a STAT3 gene signature [42].

AHR ligands are known to exert different immune-modulating effects at different dosages. Ehrlich et al. [43] compared different AHR ligands in an acute alloresponse model: FICZ has dose-dependent immune-modulating effects; 50 µg/kg FICZ promoted, while 10 mg/kg FICZ inhibited Th17 cells; a single low dose (10 mg/kg or 40 mg/kg) ITE’s effect peaked at 4hr and diminished at 20 h. Two doses of 80 mg/kg ITE administered 12 h apart or 40 mg/kg administered every 6hr induced CYP1A1 to a comparable level of 15 µg/kg TCDD, gauged by both activation of CYP1A1 transcription and inhibition of splenic CD8+T cells. In the EAE or experimental colitis model, low-dose ITE (10 mg/kg) suppressed immunity via inducing tolerogenic DCs and Tregs [44]. In our glioma model, a significantly higher dosage (100 mg/kg) was applied every other day.

Other factors, such as AHR’s interaction partners, might also contribute to ITE’s opposite immune effects. For example, estrogen receptors modulate AHR’s activity by direct interaction, and play opposite roles in glioma or EAE: The prevalence of astrocytoma is higher in men [45], while that of multiple sclerosis (modeled by EAE) is higher in women [46,47,48], suggesting that estrogen-ER activity is protective in glioma and detrimental in EAE. AHR also interacts with other factors including HIF1α, RelA, RelB, E2F1, Rb, and AR, and these factors could contribute to the opposite effects of ITE [49].

Due to the different and sometimes opposite effects of the same AHR ligand, AHR ligands have been proposed as selective modulators or rapidly metabolized ligands instead of agonists or antagonists [50,51]. Using an ultrasensitive reporter, Hoffman et al. classified AHR ligands into two classes: potent AHR activators with sustained transcriptional activation and toxic responses and mild AHR modulators with transient transcriptional conditioning and subtle therapeutic effects [52]. ITE fitted better as a mild AHR modulator. Our RNA-seq data collected 24 h after adding ITE to cell culture also supported ITE as an AHR modulator, as CYP1A1 or other cytochrome p450 gene expression changes were not detected.

Notably, other AHR ligands present in the tumor microenvironment, such as indole-derived metabolites, can bind to AHR and influence immune responses. In this study, we focused exclusively on the interaction between ITE and kynurenine, highlighting the need for further research to comprehensively elucidate AHR-mediated regulation of tumor immunity. Additionally, caution must be exercised when extrapolating findings from mouse glioma models to GBM patients, as there are significant differences in the AHR pathway between mice and humans.

## 4. Materials and Methods

### 4.1. Cell Lines, Animals and Reagents

GL261 mouse glioma cell lines were kindly provided by the Third Military Medical University. The cells were cultured in DMEM/F12 medium (Gibco, 8121048, Shanghai, China), supplemented with 10% FBS (Tianhang, 22011-8612, Hangzhou, China). Six- to eight-week-old C57BL/6J wild-type female mice were purchased from Beijing Vital River Laboratory Animal Technology (Beijing, China). All animal experiments were conducted in accordance with protocols approved by the animal care guidelines of the Inner Mongolia Agricultural University. The PD1 antibody (BioXCell, BE0273, Shenzhen, China) and other antibodies used in this study are listed in Table 1.

### 4.2. Mouse Orthotopic Glioma Model

Mice were anesthetized with an intraperitoneal injection of 0.3% Pentobarbital sodium (30 mg/kg). For the stereotactic intracranial injection, the surgical site was shaved and prepared with iodine. A midline incision was made to expose the bregma point, 1 × 10^5^ GL261 cells in a volume of 5 µL medium were stereotactically injected into the left striatum defined by the following coordinates: 2 mm posterior to the coronal suture, 2 mm lateral to the sagittal suture, and 3mm deep to the cortical surface, using a microinjection pump (KD scientific, 78-1311Q, Holliston, MA, USA) at a speed of 0.5 µL /min. The needle was removed slowly and the skin was sutured with nylon thread.

### 4.3. Drug Treatment

The mice were randomly divided into four groups, each comprising eight mice: control group, ITE group, anti-PD1 monoclonal antibody treatment group (PD1 group), and ITE plus anti-PD1 monoclonal antibody group (ITE+PD1 group). The anti-PD1 monoclonal antibody was administered twice by intraperitoneal injection, with each injection containing 100 µg and a three-day interval between them. ITE was dissolved in corn oil containing 10% DMSO and given at 100 mg/kg by intraperitoneal injection every other day. For the ITE+PD1 group, 100 mg/kg ITE was administered twice, with a one-day interval between doses, followed by a single 100 µg PD1 dose. The control group was given the same volume of 5% DMSO solution as the ITE group at the same schedule.

### 4.4. Animal Imaging

To enable in vivo imaging of the glioma, IR783 dye was used [53]. Mice with orthotopic tumors were injected with IR-783 intraperitoneally at a dose of 50 nM/mouse, 24 h before imaging. Imaging was conducted on an IVIS Lumina XR Imaging System (PerkinElmer, Waltham, MA, USA) equipped with fluorescent filter sets (excitation/emission, 783/840 nm). During image acquisition, automatic background fluorescence subtraction was performed to determine the total dye uptake in tumors, as previously described. Imaging was conducted on days 11, 14, 17, 20, 23, and 26 after tumor implantation. To ensure animal welfare, mice were euthanized when the posterior tumor edge approached the tragus of the ears, to ensure that the tumor size was well below 20 mm. Survival was analyzed using IBM SPSS 20.0 software with Kaplan–Meier analysis. For tumor growth analysis, all animals in each control or treatment arm were imaged, and tumor volume data were analyzed using the TumorGrowth online tool.

### 4.5. Preparation of Single-Cell Suspension

To prepare a single-cell suspension for flow cytometry analysis, tumors were cut into pieces and digested with Collagenase IV (200 U/mL) and DNase I (100 mg/mL) at 37 °C for 30 min. The tissue was then passed through a 70 µm cell strainer (Falcon, Shanghai, China) and washed with PBS buffer before proceeding with antibody-mediated staining.

### 4.6. Flow Cytometry

Flow cytometry experiments were conducted using a Beckman CytoFLEX S (Brea, CA, USA) flow cytometer, and data were analyzed using Beckman CytoFLEX S software (CytExpert 1.3.0.12). Cell debris was excluded using a forwarding versus side scatter plot, and doublets were excluded using a scattering height versus forward scatter area plot. Nonviable cells were excluded using Live/Dead Aqua (Invitrogen, Shanghai, China) staining. Fluorescent compensation experiments were performed by staining each antigen separately to generate the compensation matrix.

For multi-color immune cell analysis, a single-cell suspension was obtained from each brain tumor sample and stained with Aqua Live/Dead viability dye (Biolegend) in accordance with the manufacturer’s instructions. Cells were then incubated in blocking solution TruStain FcX™ (anti-mouse CD16/32) Antibody (Biolegend 101320) in PBS and stained with a standard panel of immunophenotyping antibodies in staining buffer (see Table 1 for a list of antibodies, fluorochromes, manufacturers, and concentrations) for 30 min at 4 °C. After staining, cells were washed with PBS, fixed in a 1:3 fixation/permeabilization concentrate: diluent mixture (BD) for 15 min, centrifuged, washed, and then stained for FoxP3 and IL17 in permeabilization buffer for 30 min at 4 °C. Cells were then centrifuged at 450 g, and the pellet was resuspended in the staining buffer before loading into the flow cytometer.

For MDSC induction analysis, single cells were collected from normal mouse peripheral blood and stained with each antibody separately before being analyzed using the flow cytometer.

### 4.7. Immunohistochemistry (IHC)

The brains were extracted from the mice and fixed with 4% paraformaldehyde, followed by sucrose gradient dehydration (15%, 20%, 30%), with each gradient dehydration carried out for 12 h at 4 °C. The brain sample was then embedded by OCT, frozen in liquid nitrogen, and sectioned at −25 °C, 12 µm thickness. The slices were treated in 3% hydrogen peroxide for 15 min at room temperature, washed with PBS for three times, incubated with normal goat serum at room temperature, and then incubated with rabbit anti-mouse CD4 antibody and rabbit anti-mouse CD8 primary (Bioss, Beijing, China; 1:100, 4 °C overnight) antibodies. They were washed with PBS, stained with HRP-conjugated secondary antibody, and incubated in DAB solution for color development. Four images were captured for each tissue section, and the mean number of positive cells (number/mm^2^) was calculated by using IPP 6.0 software. The criterion for positive was that the cytoplasm has brown particles.

### 4.8. Immunofluorescence

The tissue sections were fixed in 4% paraformaldehyde solution for 15 min and washed with distilled water for 5 min. Then, blocked with 10% goat serum at 28 °C for 1h. After blocking, the sections were incubated with primary antibody overnight at 4 °C, and washed 3 times with PBS buffer, 5 min each time. Then, the sections were incubated with different fluorescently labeled secondary antibodies at 37 °C for 1 h, washed with PBS buffer, and stained with DAPI solution for 15 min followed by PBS wash. The sections were then mounted with glycerol and covered with coverslips.

### 4.9. RT-PCR

Four hundred nanograms of total RNA were employed for cDNA synthesis using the ProtoScript II First Strand cDNA Synthesis kit (TaKaRa, #RR036A, Dalian, China) according to the manufacturer’s protocol. Forty nanogram cDNA was used as templates for RT-PCR using SYBR® Premix Ex Taq kit (TaKaRa, #RR820A) using LightCycler 96 (Roche, Basel, Switzerland). The sequences and efficiency of all primers were listed in Table 2, and HPRT/GAPDH was used as reference genes.

### 4.10. Western-Blot

Protein was extracted on ice in RIPA solution (Solarbio, #R0020, Shanghai, China), and quantified using a bicinchoninic acid protein assay kit (Solarbio, #PC0020). Forty microgram protein was separated by 8% SDS/PAGE and transferred to nitrocellulose membranes (MILLIPORE, #HATF00010, Shanghai, China) at 120 v for 90 min. Membranes were blocked with 5% Skim Milk solution (Solarbio, #D8340) for 3 h at 37 °C, immunoblotted with primary antibody (listed in Table 1) overnight at 4 °C, washed, and then blotted with a secondary antibody; they were washed and imaged using ODYSSEY CLX (Clx-0519, LI-COR, Gene Company Limited, Lincoln, NE, USA, Image Studio Ver5.2).

### 4.11. Mouse Peripheral Blood Mononuclear Cell Collection

(1) Anesthetize the mice with 3% chloral hydrate, take 700 µL of whole blood from the mouse heart inject an anticoagulation tube containing heparin sodium, and mix well after adding the same amount of Hank’s solution;
(2) Add 1/2 blood volume of the peripheral blood mononuclear cell separation solution (Solarbio #P6340) to the centrifuge tube, suck the mouse’s whole blood with a pipette, then slowly inject it into the liquid surface of the stratification solution along the tube wall;
(3) Put it in a centrifuge at 1500 rpm for 10 min. It can be seen that most of the albuginea-like mononuclear cells are suspended at the interface between the plasma and the stratified fluid;
(4) Suck the white membrane cell layer with a straw, transfer it to a new centrifuge, add the same amount of Hank’s solution, mix the liquid, put it in a horizontal centrifuge at 1000 rpm for 10 min, and discard the supernatant;
(5) Repeat step 4 twice;
(6) Add RPMI-1640 complete medium (1% penicillin-streptomycin solution + 10% FBS) to the pellet, mix well, transfer it to a 5 mL cell culture flask with complete medium, and cultivate it in a CO_2_ incubator.

### 4.12. Statistical Analysis

Statistical analysis was carried out by both the SPSS package and the Prism6.0 software package. Tumor growth analysis was performed by applying a linear mixed model using an online tool TumorGrowth (https://kroemerlab.shinyapps.io/TumGrowth/, accessed on 24 December 2021). Immune cell percentage in four groups was determined by ANOVA using Graphpad (version number Prism 8.0.2). Overall survival was defined as the time from glioma cell engraftment until the sacrifice of the animals. Survival curves were plotted using the Kaplan–Meier method and compared by log-rank test using SPSS (version number 26.0.1). A *p*-value less than 0.05 was considered significant.

### 4.13. Institutional Review Board Statement

The study was conducted according to the guidelines of the Declaration of Helsinki and approved by the Institutional Review Board (or Ethics Committee) of Inner Mongolia Agricultural University.

## Figures and Tables

**Table 1:** Primary antibody list.

Antibody	Brand
CD8	Abcam (Shanghai, China)
CD4	Abcam
STAT3	Abclonal (Wuhan, China)
STAT3 phosphorylation	SAB (Shanghai, China)
LY6G	CST (Shanghai, China)
Gr	CST
IL6	Abclonal
IL11	Abclonal
β-actin	Protein-tech (Wuhan, China)
GAPDH	Protein-tech
a-tubμlin	Protein-tech
Goat anti-mouse fluorescent secondary antibody-488	Abcam
Goat anti-mouse fluorescent secondary antibody-633	Abcam
Goat anti-rabbit fluorescent secondary antibody	Abcam
Goat anti-mouse fluorescent secondary antibody-800W	Li-COR Bioscience (Lincoln, NE, USA)
Goat anti-rabbit fluorescent secondary antibody-800W	Li-COR Bioscience
7-AAD	Bio-Legend (Shanghai, China)
CD45	Bio-Legend
CD3	Bio-Legend
CD4	Bio-Legend
CD8	Bio-Legend
CD25	Bio-Legend
IL17A	BD (Shanghai, China)
FOXP3	BD
LY6G	Bio-Legend
CD11C	Bio-Legend
IA/IE	Bio-Legend
LY6C	Bio-Legend
NPK46	Bio-Legend
CD11B	Bio-Legend
CD24	Bio-Legend
CD64	Bio-Legend

**Table 2:** Primer list.

Gene	Forward Primer	Reverse Primer
PTK2 (human)	ACATTATTGGCCACTGTGGATGAG	GGCCAGTTTCATCTTGTTGATGAG
HPRT (human)	TGACACTGGCAAAACAATGCA	GGTCCTTTTCACCAGCAAGCT
MYH9 (human)	ATCTCGTGCTATCCGCCAAG	GTTGTACGGCTCCAACAGGA
Myh9 (mouse)	ACGCCAAGACGGTGAAGAAT	CTTGGCGGATAGCACGAGAT
COCL1 (human)	TCTGCGACAACGGCAAGGTG	GACGCCGGTGGTTTCTTGGT
ITGA5 (human)	GCCTGTGGAGTACAAGTCCTT	AATTCGGGTGAAGTTATCTGTGG
ITGB5 (human)	CAGGTGGAGGACTATCCTGTG	GTGCCGTGTAGGAGAAAGGAG
Vav3 (mouse)	TTACACGAAGATGAGTGCAAATG	CAACACTGGATAGGACTTTATTCATC
VAV3 (human)	ACGGACCAATGGACTGCG	TTCTGCCCTGCCAAAACA
Gapdh (mouse)	AACTTTGGCATTGTGGAAGG	GGATGCAGGGATGATGTTCT
GAPDH (human)	TCATTGAGCCCTTCCACAATG	GGTGTGAACCACGAGAAATATGAC

## Data Availability

The RNAseq data have been deposited as a bioproject (https://www.ncbi.nlm.nih.gov/bioproject/?term=PRJNA789328, submitted on 15 December 2021).
